# The Explore2–2022 climate projections dataset for impact studies over France

**DOI:** 10.1016/j.dib.2026.112659

**Published:** 2026-03-09

**Authors:** Paola Marson, Jean-Michel Soubeyroux, Lola Corre, Raphaëlle Samacoïts, Eric Sauquet, Yoann Robin, Mathieu Vrac

**Affiliations:** a Direction de la Climatologie et des Services Climatiques, Météo-France, 42 Avenue Gaspard Coriolis 31100 Toulouse, France; b Météo-France, CNRS, Univ. Toulouse, CNRM, 42 Avenue Gaspard Coriolis 31100 Toulouse, France; c UR RiverLy, INRAE, Villeurbanne, France; d Laboratoire des Sciences du Climat et de l’Environnement, CEA/CNRS/UVSQ, Univ. Paris-Saclay, Institut Pierre Simon Laplace 91191 Gif-sur-Yvette, France

**Keywords:** Regional climate model, Euro-cordex, Climate model ensemble, Storylines, DRIAS, Adaptation, Hydrology

## Abstract

Within the Explore2 national project, a new set of bias-corrected regional climate projections sub-sampled from the EURO-CORDEX (EUR11) ensemble has been produced to describe the impact of climate change on water resources and to support impact studies over mainland France. This dataset has been specially selected to reflect the expected changes in temperature and precipitation of the complete EURO-CORDEX (EUR11) ensemble while taking those of CMIP6 into account as consistency constraint. Yet, the selection allows to obtain a smaller ensemble size to handle with. The process of GCM/RCM couples selection is fully described in the article. The dataset makes it possible to characterize and partition the various sources of uncertainty about the evolution of the climate in France, by taking into account three greenhouse gas emission scenarios (RCP 2.6, RCP 4.5 and RCP 8.5), multiple regional climate models (allowing to dispose to 9 to 17 GCM/RCM couples depending on the emission scenario), two methods of statistical bias correction (ADAMONT and CDF-t) and continuous time series to explore internal variability.

This dataset contains 10 climate variables at daily resolution, enabling the calculation of a very large number of climate impact indicators, as well as its use to drive a wide variety of hydrological models in France. Examples of climate change representations suitable for this dataset are provided for cumulative precipitation at seasonal scale. These representation methods are intended to guide potential users of this data when aiming to characterize the robustness of the changes (according to individual simulations, time horizons or climate change scenarios) and to identify contrasting scenarios across a territory. A narrative approach is also proposed to facilitate the exploration of individual projections of climate change, allowing for a more accurate consideration of inter-annual variability and extremes Four narratives were selected among the 17 GCM/RCM couples in collaboration with hydrologists which correspond to contrasting changes of temperature and precipitation in order to reflect a plurality of contrasting possible climate futures within the dispersion of the Explore2–2022 dataset.

The richness of this dataset and the inclusion of the most recent regional climate simulations for France justified its use in constructing and illustrating the reference warming trajectory for climate change adaptation (TRACC) in France, backed by the 3rd National Climate Change Adaptation Plan.

The Explore2 project worked to build a data-set meeting the FAIR Data Principles^1^ to maximize transparency, easiness and re-usability of data.

Specifications Table

**Table:** 

Subject	Earth & Environmental Sciences
Specific subject area	Bias corrected daily regional climate projections as a thoughtful sub-sample of EURO-CORDEX ensemble suitable for impact studies over France, and particularly for hydrology
Type of data	NetCDF files (.nc) containing two dimensional (lat,lon) bias corrected climate data on a Lambert conformal (regular) grid (EPSG 27572) over France comprising 143×134 = 19162 points with a horizontal resolution of 8 km
Data collection	The Explore2–2022 dataset is composed as a set of daily baseline (1951–2005) and transient future climate scenarios (2006–2100, under three emissions scenarios: RCP 2.6, RCP 4.5, RCP 8.5) from GCM/RCM pairs thoughtfully selected from the full EURO-CORDEX (EUR11) ensemble (https://euro-cordex.net/), then bias corrected over France by two statistical adjustment methods (ADAMONT, CDF-t). The selection accurately reproduces the statistical distribution of climate change signal over France of EURO-CORDEX; it respects criteria of physical plausibility of simulations; the selection is made consistent to latest CMIP6 simulations.
Data source location	Primary data are climate simulations from the EURO-CORDEX (EUR 11) experiment, then selected and bias corrected over France. https://euro-cordex.net/ There is no central EURO-CORDEX archive. Simulations are available from ESGF nodes (https://cordex.org/data-access/cordex-cmip5-data/cordex-cmip5-esgf/) as well as in the Copernicus Climate Data Store (https://cds.climate.copernicus.eu/datasets/projections-cordex-domains-single-levels?tab=overview)
Data accessibility	Repository name: IPSL Data catalog: Explore 2 dataset: a set of regionalized and bias corrected climate projections for impact studies over France. Data identification number: https://doi.org/10.14768/4aba213f-ad73–431d-b914–88ce1b42468d Direct URL to data: https://data.ipsl.fr/catalog/srv/eng/catalog.search#/metadata/4aba213f-ad73–431d-b914–88ce1b42468d
Related research article	None

## 1 Value of the Data

• Explore2–2022 [1] bias corrected regional climate data are reference national data for studies on the impacts of climate change in France and are royalty-free and easily accessible to both adaptation stakeholders and researchers on the national DRIAS^2^ portal and on ESPRI^3^ portal.
• Explore2–2022 data are a (renewed) selection of EURO-CORDEX (EUR11) regionalised climate simulations, which updates the DRIAS-2020 [2] dataset produced by Météo-France in 2020: it meets new and more severe selection criteria, employs more recent regional simulations and is consistent to new CMIP6 simulations.
• Explore2–2022 has purposefully been produced for studying climate change impacts on hydrology: the dataset can be valuably used as input for hydrological projections as they include the main variables driving hydrological processes and has the relevant time frequency for hydrological modelling. They were used as input for the hydrological projections of the national Explore2 project, which aims to characterize the future of water resource in France [3]. Yet, the data-set meets the wider scope of serving a larger spectrum of impact studies and climate services.
• The richness of the Explore2–2022 dataset justified its use in constructing the reference warming trajectory for climate change adaptation in France (TRACC) [4], backed by the 3rd National Climate Change Adaptation Plan.
• Explore2–2022 data are already being used by several climate services for adaptation in France, such as Climadiag Commune^4^ and Climadiag Agriculture^5^
• Beyond national applications, Explore2–2022 can constitute a prototype for similar efforts in other regions and countries offering both a replicable construction methodology and a framework of solid and validated applications in impact studies and climate services.

## 2 Background

With climate change having a strong impact on mainland France in recent years, socio-economic players necessitate up-to date future climate information to support impact studies and design adaptation plans. Since 2012, Météo-France constructs bias-corrected regional climate projections sets over France and makes them freely available on the national climate portal *DRIAS - les-futurs-du-climat*.^6^ The latest available set was DRIAS-2020 [2]. Within the Explore2 project (2021–2024),^7^ the new Explore2–2022 [1] set has been produced. Explore2–2022 is as a renewed selection from EURO-CORDEX^8^ (EUR 11): with respect to DRIAS-2020, new projections have been added, while others have been discarded to meet new criteria, be consistent to new CMIP6 simulations and employ more recent regional simulations.

Explore2–2022 has purposefully been produced to update knowledge on climate change impacts on water resources in France. Yet, it meets the wider scope of being a reference national dataset and serve a larger spectrum of impact studies and climate services. Scientific articles ([3,5]) and reports^9^ already characterize impacts using this data-set as climate forcing.

For preparing the third French National Climate Change Adaptation Plan, the Explore2–2022 data-set has been used to illustrate the TRACC^10^ by portraying the projected expected climate at given levels of warming over France.

## 3 Data Description

The Explore2–2022 dataset is composed of a set of 53 regional climate simulations (i) thoughtfully sub-sampled from the EURO-CORDEX (EUR11) ensemble at 12 km resolution; (ii) bias corrected by separately applying two statistical bias correction methods which downscale them on a 8 km resolution grid covering mainland France.

Each available simulation contains ten climatic variables, spanning temperature, precipitation, humidity, solar radiation, and wind speed, as well as the evapotranspiration indicator calculated from them. All of those variables are available at the daily time step and allow a large and comprehensive analysis of impacts of climate change. Table 2 lists and describes the selected and bias corrected variables available in the dataset.

The available simulations cover the 1951–2100 period (see Table 1 for more details), with data from the historical (baseline) runs on the period 1951–2005 (17 GCM/RCM pairs) and future projections covering the period 2006–2100 under three distinct emission scenarios: 17 GCM/RCM pairs under the RCP 8.5 scenario (high emissions); 9 GCM/RCM pairs under the RCP 4.5 scenario (intermediate emissions) and 10 GCM/RCM pairs under the RCP 2.6 scenario (low emissions). Table 1 presents the selected model pairs and the available scenarios for each of them.

**Table 1:** Details of Explore2 dataset content in terms of GCM/RCM pairs under RCP 2.6, RCP 4.5 and RCP 8.5 and historical runs.

	Member	RCM	Version	Historical	RCP 2.6	RCP 4.5	RCP 8.5	Period
CNRM-CERFACS-CNRM-CM5	r1i1p1	CNRM-ALADIN63	v2	X	X	X	X	19510101-21001231
CNRM-CERFACS-CNRM-CM5	r1i1p1	MOHC-HadREM3-GA7–05	v2	X			X	19520101-21001231
ICHEC-EC-EARTH	r12i1p1	KNMI-RACMO22E	v2	X	X	X	X	19500101-21001231
ICHEC-EC-EARTH	r12i1p1	SMHI-RCA4	v1	X	X	X	X	19700101-21001231
ICHEC-EC-EARTH	r12i1p1	MOHC-HadREM3-GA7–05	v1	X	X		X	19520101-21001231
MOHC-HadGEM2-ES	r1i1p1	CNRM-ALADIN63	v1	X			X	19500101-20991231
MOHC-HadGEM2-ES	r1i1p1	CLMcom–CCLM4–8–17	v1	X		X	X	19500101-2099*
MOHC-HadGEM2-ES	r1i1p1	ICTP-RegCM4–6	v1	X	X		X	19710101-20991231
MOHC-HadGEM2-ES	r1i1p1	MOHC-HadREM3-GA7–05	v1	X	X		X	19520101-2099⁎⁎
IPSL-IPSL-CM5A-MR	r1i1p1	DMI-HIRAM5	v1	X			X	19510101-21001231
IPSL-IPSL-CM5A-MR	r1i1p1	SMHI-RCA4	v1	X		X	X	19700101-21001231
MPI-M-MPI-ESM-LR	r1i1p1	CLMcom–CCLM4–8–17	v1	X	X	X	X	19500101-21001231
MPI-M-MPI-ESM-LR	r1i1p1	ICTP-RegCM4–6	v1	X	X		X	19700101-2100⁎⁎⁎
MPI-M-MPI-ESM-LR	r1i1p1	MPI-CSC-REMP2009	v1	X	X	X	X	19500101-21001231
NCC-NorESM1-M	r1i1p1	DMI-HIRAM5	v3	X		X	X	19510101-21001231
NCC-NorESM1-M	r1i1p1	GERICS-REMO2015	v1	X	X	X	X	19500101-21001231
NCC-NorESM1-M	r1i1p1	IPSL-WRF381P	v1	X			X	19510101-21001231

⁎ MOHC-HadGEM-ES/CLMcom-CCLM4–8–17 RCP4.5 19500101–20991130. *MOHC-HadGEM-ES/CLMcom-CCLM4–8–17 RCP8.5 19500101–20991231.

⁎⁎ MOHC-HadGEM-ES/MOHC-HadREM3-GA7–05 RCP2.6 19520101–20991229. ^⁎⁎^MOHC-HadGEM-ES/MOHC-HadREM3-GA7–05 RCP8.5 19520101–20991219.

⁎⁎⁎ MPI-M-MPI-ESM-LR/ICTP-RegCM4–6 RCP2.6 19700101–21001130. ^⁎⁎⁎^MPI-M-MPI-ESM-LR/ICTP-RegCM4–6 RCP8.5 19700101–21001230.

Each simulation has been separately bias-corrected with two statistical methods: ADAMONT [6] and CDF-t [7]. Both methods were applied having the SAFRAN reanalysis [8] as reference data; thus, while correcting them, the two methods downscale the simulations onto the SAFRAN 8 km resolution grid. Details on the grid are given below (see “Horizontal grid” paragraph within this section). With each simulation being corrected and downscaled twice, the dataset provides two bias-corrected runs for each GCM/RCM selected couple, which allows to account for the uncertainty coming from the statistical method applied. G. Evin [5] assesses the overall uncertainty components of the ensemble by the QUALIPSO method. The sources of uncertainties vary according to regions and are not the same for different climate indicators. As found in G. Evin [5], for temperature projections, the emission scenario is the main contribution for all seasons. A non-negligible contribution of climate models is found in summer. It mostly comes from GCM in coastal areas and from RCM in mountainous areas. For summer precipitation, the main contribution comes from RCMs, then from emission scenarios; in winter, the main contribution comes from both GCM and RCM in coastal and mountainous regions. The uncertainty contribution of each model category increases with projection time. The contribution due to bias correction methods for temperature and precipitation is found to be much smaller than that of the other sources of uncertainty. Internal variability can be non-negligible, especially for precipitation. It is likely to cause substantial deviation from the climate response, potentially leading to unusual or critical years or sequences of years. The reader is encouraged to see G. Evin [5] for a very comprehensive analysis of the various components of total uncertainty linked to this data set, as well as in its hydrological applications.

Corrections obtained by the two statistical methods are of equal value and usability. More insights on the procedure of bias correction are provided in the section “Experimental design, materials and methods”.

Table 2 lists and describes the selected and bias corrected variables available in the dataset. Please refer to the paragraph “Bias correction of precipitation and snowfall” of the section “Experimental design, materials and methods” for details on the statistical bias correction of snowfall.

**Table 2:** List of available variables in the Explore2–2022 data-set.

	Unit	Standard Name	Long name
hussAdjust	$kg\cdot kg^{-1}$	specific_humidity	Near Surface Specific Humidity
prtotAdjust	$kg\cdot m^{-2}\cdot s^{-1}$	precipitation_flux	Total Precipitation Flux*
rldsAdjust	$W\cdot m^{-2}$	surface_downwelling_longwave_flux_in_air	Surface Downwelling Longwave Radiation
rsdsAdjust	$W\cdot m^{-2}$	surface_downwelling_shortwave_flux_in_air	Surface Downwelling Shortwave Radiation
sfcWindAdjust	$m\cdot s^{-1}$	wind_speed	Near-Surface Wind Speed
tasmaxAdjust	K	air_temperature	Daily Maximum Near-Surface Air Temperature
tasminAdjust	K	air_temperature	Daily Minimum Near-Surface Air Temperature
tasAdjust	K	air_temperature	Daily Average Near-Surface Air Temperature
prsnAdjust	$kg\cdot m^{-2}\cdot s^{-1}$	snowfall_flux	Snowfall Flux*
evspsblpotAdjust	$kg\cdot m^{-2}\cdot s^{-1}$	water_potential_evapotranspiration_flux	Potential evaporation* including sublimation and transpiration

⁎ To convert those fluxes to daily cumulated quantities (mm/day), apply a factor 86400 (seconds in a day).

### 3.1 Horizontal grid

All data is available in NetCDF format on a Lambert conformal (regular) grid (EPSG 27572^11^) over France comprising 143×134 = 19162 points with a horizontal resolution of 8 km. Only 8981 points (∼47 %) correspond to land points on the French territory and have non-NA data. Table 3 reports the attributes of the NetCDF files which describe the grid (Table 4).

**Table 3:** Attributes of the NetCDF files on Lambert Paris II grid.

	Attribute	Value
x	units	m
	long_name	X coordinate of projection
	standard_name	projection_x_coordinate
y	units	m
	long_name	Y coordinate of projection
	standard_name	projection_y_coordinate
lat	axis	y
	long_name	Latitude coordinate
	standard_name	latitude
	units	degrees_north
lon	axis	x
	long_name	Longitude coordinate
	standard_name	longitude
	units	degrees_east
LambertParisII	grid_mapping_name	lambert_conformal_conic_1SP
	epsg	27572

**Table 4:** Elements composing the name of the files.

	Description
variable	Bias corrected variable. See Table 2
zone	France
GCM	The forcing GCM. See Table 1
scenario	The emission scenario. It takes values historical, rcp26, rcp45, rcp85
member	The member. See Table 1
RCM	The nested RCM. See Table 1
version	The experiment version. See Table 1
bc-id	Identification of the bias correction method. It takes values MF-ADAMONT-SAFRAN–1980–2011 or LSCE-IPSL-CDFt-L-1V-0L–1976–2005. See section on bias correction
timestep	day
timeperiod	The period of time covered by the simulation. Example 19510101 - 20991231. See Table 1.
suffix	A suffix might be necessary, as for evspsblpotAdjust indicating the formula used for calculation. See paragraph about *potential evapotranspiration*.

### 3.2 Name of files

The names of netCDF files have the following format:

variable_zone_GCM_scenario_member_RCM_version_bc-id_timestep

_timeperiod_suffix.nc

For example, the netcdf file containing the daily maximum temperature, corrected using the ADAMONT method, from the ICHEC-EC-EARTH/KNMI-RACMO22E pair for the historical period is named:

tasmaxAdjust_France_ICHEC-EC-EARTH_historical_r12i1p1_KNMI-RACMO22E

 _v1_MF-ADAMONT-SAFRAN-1980–2011_day_19500101–20051231.nc.

Please note that the zone and the timestep fields are fixed for all files (respectively “France” and “day”)

### 3.3 The dataset representation and content

The Explore2–2022 dataset enables the extraction of climate information and the calculation of climate indicators; by way of example, and in order to illustrate the content of the dataset, we will display the projected evolution of seasonal precipitation in the 21st century, as indicators of the primary drivers of the evolution of water resources in France.

Their evolution is displayed at three time horizons, H1: 2021–2050, H2: 2041–2070, H3: 2070–2099, with respect to the baseline period 1976–2005.

**Fig. 1:**
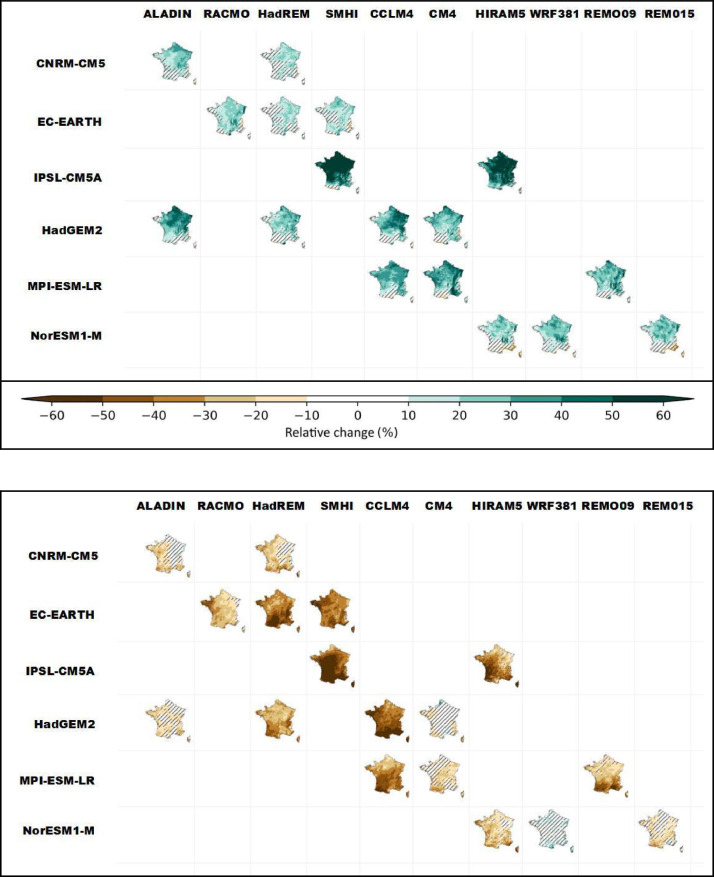
*Ensemble matrices* displaying the maps of relative differences ( %) in cumulative winter (DJF) (top panel) and summer (JJA) (bottom panel) precipitation at the end of century (H3, 2070–2099) for the RCP 8.5 scenario compared with the 1976–2005 reference period for each of the 17 GCM/RCM pairs in the Explore2–2022 ensemble. The rows correspond to GCMs, the columns to RCMs (ensemble Explore2–2022, data corrected by ADAMONT method). Hatching marks the grid points where the changes are non-significant with respect to the internal variability, according to the method applied in IPCC AR6.

**Fig. 2:**
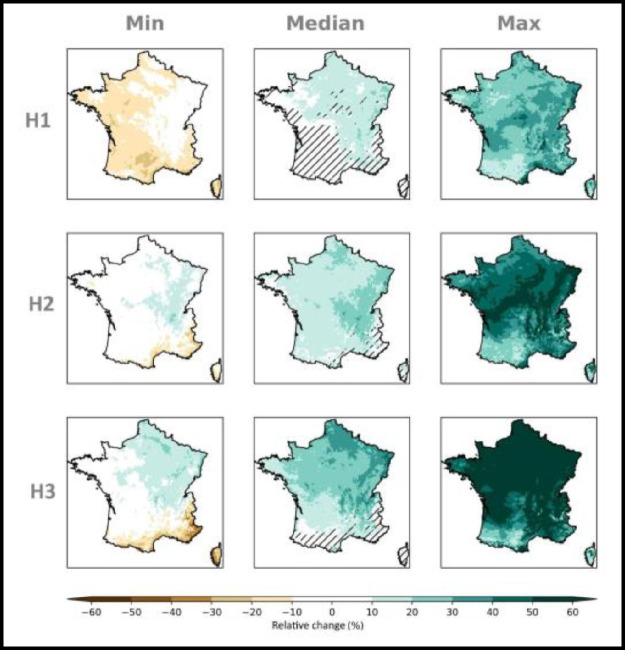
*multi-horizon* matrix displaying the spatialized distribution of the relative changes (%) in winter (DJF) precipitation within the ensemble Explore2–2022 at three time horizons, H1: 2021–2050, H2: 2041–2070, H3: 2070–2099 with respect to the baseline period 1976–2005 under the RCP 8.5 scenario. At each grid point the minimum (left column), median (central column) and maximum (right column) value within the ensemble is shown. Hatches on the map of median changes is when for a grid point the accord in sign of the 80 % of simulations is not reached. Data displayed is corrected by the ADAMONT method.

**Fig. 3:**
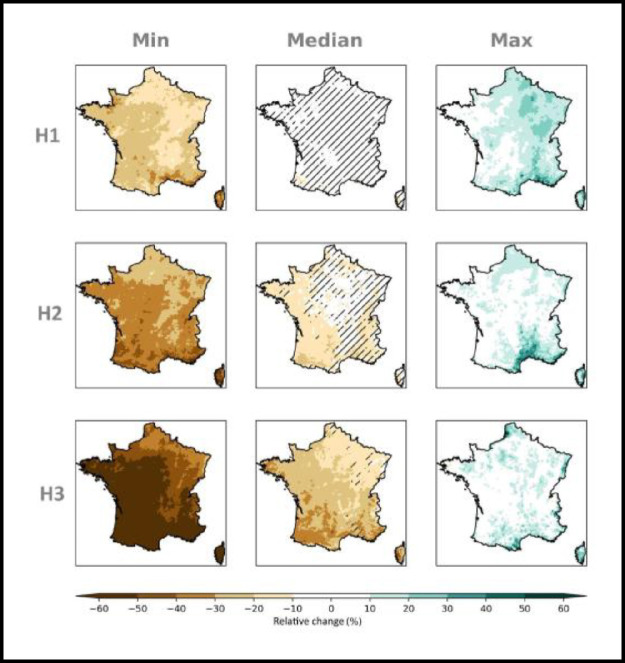
*multi-horizon* matrix displaying the spatialized distribution of the relative changes (%) in summer (JJA) precipitation within the ensemble Explore2–2022 at three time horizons, H1: 2021–2050, H2: 2041–2070, H3: 2070–2099 with respect to the baseline period 1976–2005 under the RCP 8.5 scenario. At each grid point the minimum (left column), median (central column) and maximum (right column) value within the ensemble is shown. Hatches on the map of median changes is when for a grid point the accord in sign of the 80 % of simulations is not reached. Data displayed is corrected by the ADAMONT method.

**Fig. 4:**
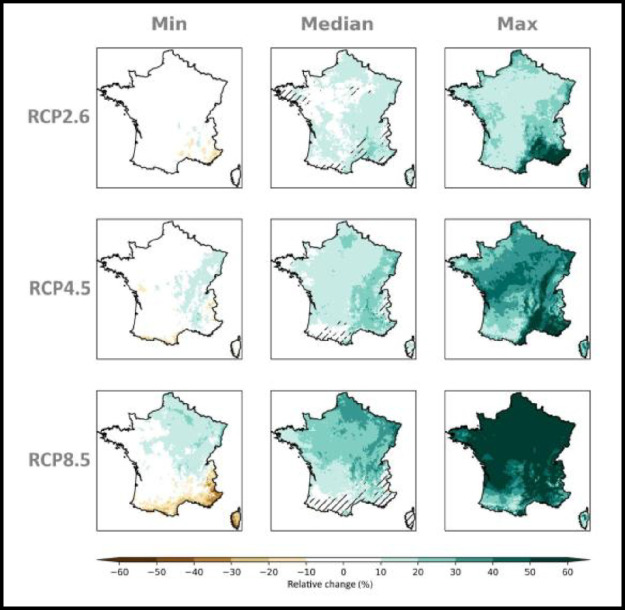
*multi-scenario* matrix displaying the spatialized distribution of the relative changes (%) in winter (DJF) precipitation within the ensemble Explore2–2022 at the time horizon H3: 2070–2099 with respect to the baseline period 1976–2005 under the emission scenarios RCP 2.6, RCP 4.5 and RCP 8.5. At each grid point the minimum (left column), median (central column) and maximum (right column) value within the ensemble is shown. Hatches on the map of median changes is when for a grid point the accord in sign of the 80 % of simulations is not reached. Data displayed is corrected by the ADAMONT method.

**Fig. 5:**
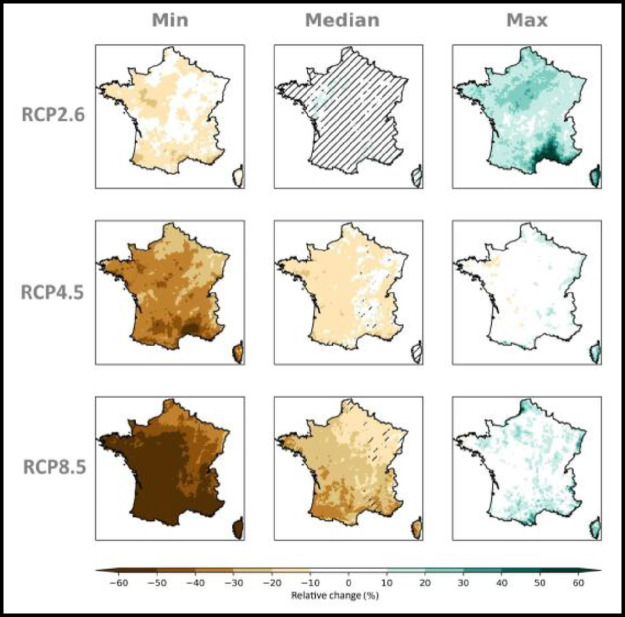
*multi-scenario* matrix displaying the spatialized distribution of the relative changes (%) in summer (JJA) precipitation within the ensemble Explore2–2022 at the time horizon H3: 2070–2099 with respect to the baseline period 1976–2005 under the emission scenarios RCP 2.6, RCP 4.5 and RCP 8.5. At each grid point the minimum (left column), median (central column) and maximum (right column) value within the ensemble is shown. Hatches on the map of median changes is when for a grid point the accord in sign of the 80 % of simulations is not reached. Data displayed is corrected by the ADAMONT method.

**Fig. 6:**
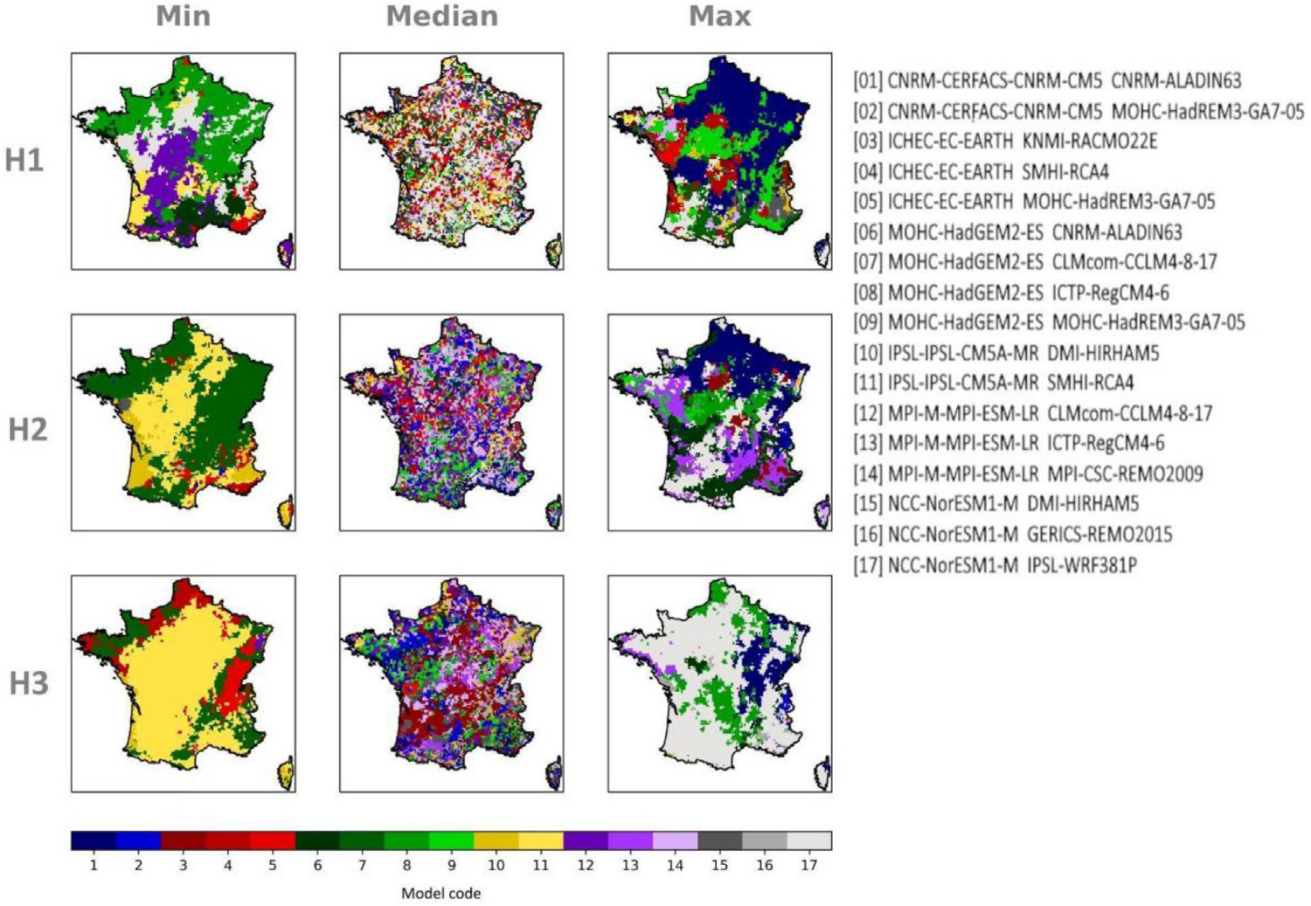
*mosaic matrices* referring to the summer (JJA) precipitation evolution displayed in the multi-horizon matrix in Fig. 3.

Some graphical representations, as those proposed in Figs. 1–6, help display the behavior of the chosen indicators within the ensemble and part of the scientific content of the dataset, in order to illustrate its value and suggest potential uses. Besides these graphical representations, which encompass the complexity of the ensemble and the variability within, a storyline approach (see the paragraphs on the “Storyline approach” within this section) is also proposed as a means for the user to follow single paths of evolution by focusing on individual simulations selected in an expert manner.

An analysis on a wider set of indicators as well as more details on the use of climate indicators from the Explore2–2022 dataset are available on [9].

E*nsemble matrices* allow to simultaneously map the evolution of an indicator among all the GCM/RCM pairs of the ensemble for a given time horizon and emission scenario. Fig. 1 illustrates the cumulated winter (DJF) and summer (JJA) precipitation over France, under RCP 8.5 and at the end of the century (H3: 2070–2099) as a relative change from the baseline period 1976–2005. Hatching marks the grid points where the changes are non-significant with respect to the internal variability, according to the method applied in IPCC AR 6. In winter, the signal of increased winter precipitation predominates in all simulations, with uncertainties about the robustness of the change being very frequent in the south and west of the country. Conversely, in summer, the signal of decreased precipitation is generally present, with uncertainties about the robustness of the change being very unevenly distributed across simulations.

Maps in *multi-scenario* or *multi-horizon* matrices can be used to synthesize the expected changes within the ensemble Explore2–2022. *multi-horizon* matrices are composed of 9 maps displaying the expected changes of a climate indicator under a given RCP scenario at different time horizons (e.g. H1: 2021–2050, H2: 2041–2070, H3:2070–2099) with respect to a baseline period 1976–2005. At each grid point on the maps, this representation shows the distribution of changes within the ensemble: in the left column the maps report the minimum change projected by the ensemble, in the central column the maps report the median projected change within the ensemble and maps in the right column report the maximum projected change within the ensemble. Fig. 2 and Fig. 3 are two examples of this representation for respectively winter and summer precipitation projected evolution under the RCP 8.5 scenario. Hatches on the map of median changes is when for a grid point the accord in sign of the 80 % of simulations is not reached.

On the contrary, *multi-scenarios* matrices, display the distribution of projected changes at a given horizon (e.g. H3:2070–2099) with respect to a baseline period (1976–2005), under the three available emissions scenarios. Fig. 4 and Fig. 5 are examples of this kind of representation for relative changes in respectively winter DJF and summer JJA precipitation at H3: 2070–2099 with respect to the baseline period 1976–2005 under the RCP 2.6, RCP 4.5 and RCP 8.5. Hatches on the map of median changes is when for a grid point the accord in sign of the 80 % of simulations is not reached.

Accompanying the multi-horizon and multi-scenario matrices, *mosaic* representations show to which model belong the displayed statistics (minimum, median or maximum projected change) on each grid point in the associated multi-horizon or multi-scenario matrix. This information is very useful for water managers who need to identify contrasting climate simulations in their territory.

Fig. 6 constitutes an example of mosaic representation in correspondence to the multi-horizon matrix of Fig. 3. Each color and code-number identifies a model, with primary color identifying a GCM and color nuances the couple they form with their nested RCM.

Analogous figures for data corrected by the CDF-t method are provided in supplementary material. See supplementary material, Figs. 1–6.

Fig. 1 suppl. shows *ensemble matrices* displaying the maps of relative differences (%) in cumulative winter DJF (top panel) and summer JJA (bottom panel) precipitation at the end of century (H3, 2070–2099) for the RCP 8.5 scenario compared with the 1976–2005 reference period for each of the 17 GCM/RCM pairs.

Figs. 2 and 3 suppl. are *multi-horizon* matrices displaying the spatialized distribution of the relative changes in winter and summer precipitation within the ensemble.

Figs. 4 and 5 suppl. are *multi-scenario* matrices displaying the spatialized distribution of the relative changes in winter and summer precipitation at the end of the century with respect to the three emission scenarios within the ensemble.

No notable differences exist between those pairs of figures: the sign, the intensity of the signal and the spatial distribution of changes are comparable between the two bias-correction methods. That is true either looking at individual models, (as in *ensemble matrices*), or in multimodel representations (as in *multi-horizon* and *multi-scenario* matrices).

Fig. 6 suppl. is a *mosaic matrix* referring to summer precipitation evolution displayed in the *multi-horizon* matrix in Fig. 3 suppl. This analysis also reveals significant similarities in the contribution of individual models to the median and maximum statistics, regardless of the time horizon with respect to those found on ADAMONT corrections. However, there are some differences in the contribution to the minimum statistic: two models contribute significantly to this statistic (MOHC-HadGEM2-ES/CCLMcom-CCLM4–8–17 and IPSL-IPSL-CM5A-MR/SMHI-RCA4) but in different proportions over the French territory depending on the two correction methods. This is consistent with results in Fig. 8.

### 3.4 Storyline approach

Providing actionable information on possible future climate scenarios involves synthesizing climate projections that present contrasting, even contradictory, future changes. The ‘probabilistic’ approach describes future changes using statistics (average or median of simulations, quantiles, etc.). To facilitate the use of projections in impact studies, an alternative known as the ‘narrative’ approach has been proposed [10]. A narrative is defined as a physically coherent sequence of past or future events. This approach seeks to develop descriptive ‘stories’ of possible future climates [10]. Several narratives must be considered in order to explore several possible futures. In addition, it is important to describe how they were selected and how they fit within the distribution of all models.

Within this Explore2–2022 dataset, four narratives were selected in collaboration with hydrologists to illustrate contrasting futures of water resources in mainland France [3]. This approach meets the needs of stakeholders who might not have the computational possibility to consider all simulations. The selection criteria focused on average future changes in temperature and precipitation: the four narratives have been selected to (i) correspond to contrasting changes in order to reflect a plurality of contrasting possible futures within the Explore2–2022 dataset under the RCP 8.5 scenario at the end of the century (H3: 2070–2099) with respect to the baseline period 1976–2005 while (ii) remaining consistent with the confidence interval of the CMIP6 projections (Fig. 8). They are intended to be used as input for hydro-climatic projections to characterize impacts on soil water, surface water flows and groundwater from identified contrasted climatic changes.

**Fig. 7:**
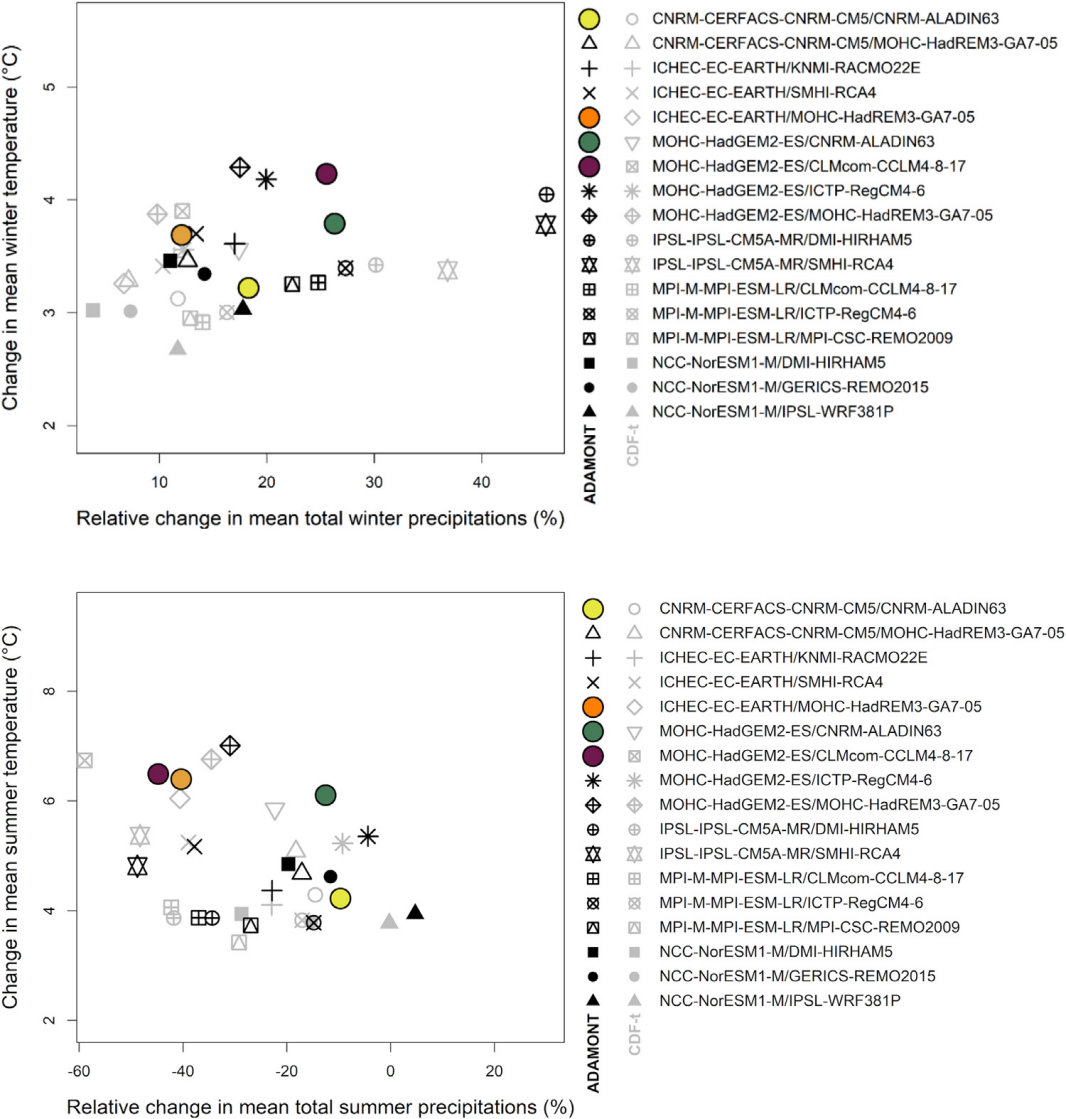
Temperature and precipitation changes in winter (DJF) and summer (JJA) in France (spatial average over land-only points) according to the 17 Explore2–2022 simulations under the RCP 8.5 scenario at the end of the century (H3: 2070–2099) with respect to the baseline period 1976–2005: The four narratives orange, green, purple and yellow are represented by colored symbols.

The selected narratives are: the **“orange narrative”** (ICHEC-EC-EARTH/MOHC-HadREM3-GA7–05): significant warming and significant drying in summer; the “**yellow narrative”** (CNRM-CERFACS-CNRM-CM5/CNRM-ALADIN63): relatively minor future changes; the “**purple narrative”** (MOHC-HadGEM2-ES/CLMcom-CCLM4–8–17): significant warming and strong seasonal contrasts in precipitation; and the “**green narrative”** (MOHC-HadGEM2-ES/CNRM-ALADIN63): significant warming and increased precipitation. Fig. 7 shows the cloud of climate models and the four highlighted narratives with corresponding colored symbols. Points in the figures refer to spatial means over land-only points. Panel 1 refers to winter (DJF) changes and panel 2 to summer (JJA) changes. The rigorous criteria used to select all the GCM/RCM couples of the Explore2–2022 set (as detailed in section “Experimental design, material and methods”) ensure the physical plausibility of these narratives. Examples of the use of these four narratives can be found in [3].

Users of this data-set can build their own criteria and select different narratives.

## 4 Experimental Design, Materials and Methods

### 4.1 Construction of the explore2–2022 dataset

The climate projections used to build the Explore2–2022 dataset are based on regional climate models simulations from the most recent EURO-CORDEX (EUR11) CMIP5 initiative.^12^ EURO-CORDEX simulations are composed of global climate models (GCM) with nested regional climate models (RCM) simulations [11]: the GCMs simulate global climate evolution while the RCMs refine the simulations over the European domain. Regional models offer a more accurate representation of precipitation and other climate variables than global models at spatial scales relevant for hydrology, as shown in particular by the Copernicus Climate Change Service at European level^13^. The EURO-CORDEX ensemble provides an exceptionally rich groundwork for climate services. Still, the projected changes are not uniformly distributed within the ensemble, nor the models are of equal quality. Moreover, the size of the complete ensemble, with over a hundred simulations available, can make the ensemble quite costly and difficult to use. To address this, we sub-sampled the whole ensemble by selecting a smaller yet robust subset that could accurately reproduce the statistical range and distribution of climate change signals over France found in the full EURO-CORDEX ensemble. Moreover, the selection is consistent with the projected changes of the latest CMIP6 simulations. We achieved this through a three-step process.

**1 First criteria list**

The Explore2–2022 dataset depends on the previous climate regional DRIAS-2020 dataset [2], namely a consistent set of 12 GCM/RCM couples from EURO-CORDEX (EURO 11) ensemble and already publicly distributed on the DRIAS portal. Eight criteria had been defined for the selection of GCM/RCM couples for the DRIAS-2020 dataset aiming at (i) having a large enough set allowing a proper understanding of the different sources of uncertainty in climate projections while (ii) optimizing the size of this set to facilitate processing by users and (iii) covering as best as possible the range of future changes in temperature and precipitation resulting from the total set for France. The eight criteria are the following:

• **Number of pairs**: around ten GCM/RCM pairs had to be selected, resulting in 12 pairs at the end of the selection process;
• **RCP** (*Representative Concentration Pathway*) scenarios availability and consistency: each pair should be available for at least two RCP scenarios:
• **GCM quality**: GCMs must be deemed realistic over Europe,^14^
• **Balance**: maximum number of different RCMs with a balanced distribution in terms of projected changes,
• **Absence of known errors**: GCM/RCM pairs affected by a known error are rejected,
• **Account for French simulations**: simulations carried out by French research institutes should be included,
• **Physical consistency**: couples with physical consistency between GCM and RCM are to be favoured,
• **Consistent spread**: the dispersion of climate change signal from the EURO-CORDEX (EUR 11) ensemble must be preserved.

The application of these criteria resulted in specifying a list of 12 GCM/RCM pairs, making a total of 30 simulations of future and past climate: 12 historical simulations (1951 to 2005); 12 RCP 8.5 projections, 10 RCP 4.5 projections and 8 RCP 2.6 projections [2]. A particular attention was paid to the criterion concerning the conservation of the dispersion: the objective for DRIAS-2020 was to ensure that the reduction in the number of simulations would result in as little loss of information as possible with respect to the full EURO-CORDEX (EUR 11) ensemble.

**2. Complementary Explore2 criteria**

Within the Explore2 project, the DRIAS-2020 ensemble has been thoughtfully enriched of new simulations from the EURO-CORDEX ensemble, in order to respond to new criteria needed in the project:

• **Evolving aerosol forcing**: we wanted the dataset to include the latest EURO-CORDEX simulations allowing evolving aerosols,
• **Redundancy**: enable the use of the QUALYPSO method [5] for uncertainty decomposition within the dataset, which requires each GCM and RCM to be present more than once.

These two new criteria brought to the addition of 7 GCM/RCM couples (listed in Table 5), thus composing an ensemble of 19 raw simulations under RCP 8.5.

**Table 5:** List of new raw simulations selected within the project Explore2 according to two new selection criteria.

GCM	RCM	RCP 2.6	RCP 4.5	RCP 8.5	Hist
CNRM‐CERFACS‐CNRM‐CM5	MOHC‐HadREM3‐GA7‐05			x	x
ICHEC‐EC‐EARTH	MOHC‐HadREM3‐GA7‐05	x		x	x
IPSL‐IPSL‐CM5A‐MR	DMI‐HIRHAM5			x	x
MOHC‐HadGEM2‐ES	CNRM‐ALADIN63			x	x
MOHC‐HadGEM2‐ES	MOHC‐HadREM3‐GA7‐05	x		x	x
MPI‐M‐MPI‐ESM‐LR	ICTP‐RegCM4‐6	x		x	x
NCC‐NorESM1‐M	IPSL‐WRF381P			x	x

**3. Consistency with CMIP6 data**

All the 19 selected projections were at this point bias corrected (see the dedicated paragraph in this section).

With new CMIP6 global simulations starting to be used for impact studies, the consistency of the Explore2–2022 regional set to the CMIP6 ensemble looked desirable, in addition to the consistency with respect to the full EURO-CORDEX ensemble. Thus, to assure this consistency, the bias-corrected simulations under RCP 8.5 have been simultaneously compared in terms of temperature and precipitation changes in the period 2070–2099 with respect to the baseline period 1976–2005 with the changes projected by the raw CMIP6 simulations under SSP5–8.5 scenario. SSP5–8.5 scenario is designed to reproduce the same radiative forcing as CMIP5 RCP 8.5 (8.5 W m⁻² by 2100) and is therefore among the CMIP6 socio-economic and emissions scenarios, the closest to the RCP 8.5 used in CMIP5. Changes in summer and winter seasons were separately considered. For temperatures, we also compared the 19 couples with the changes inferred from CMIP6 projections constrained by historical global and French temperature observations over France [12], which reduces the range of uncertainty associated with future changes.

**Fig. 8:**
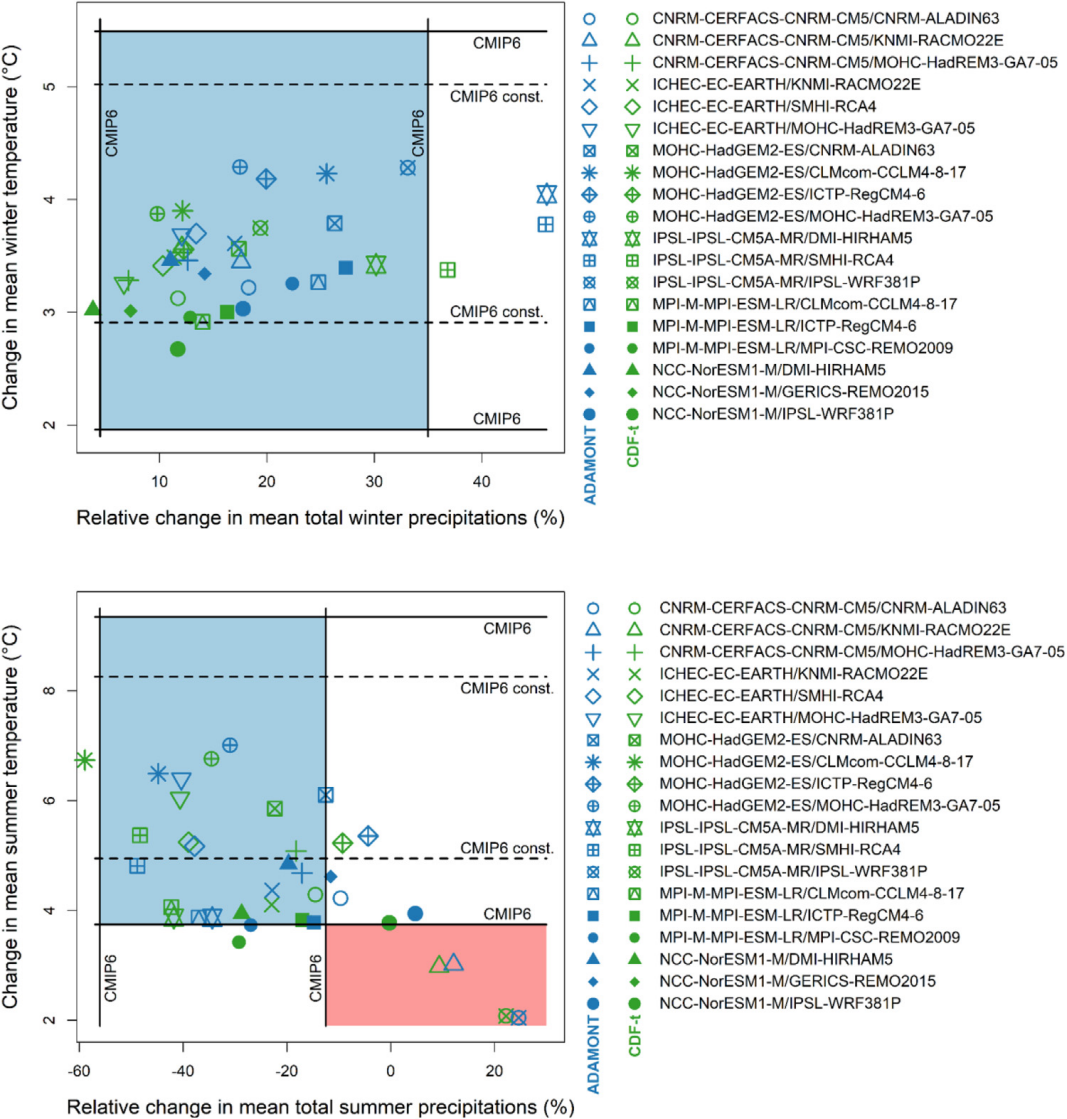
Changes in temperature and precipitation over France (spatial mean over land-only points) in the 19 Explore2 simulations corrected by the ADAMONT (blue) and CDF-t (green) methods in winter and summer seasons under the RCP 8.5 at the horizon 2070–2099 with respect to the baseline period 1976–2005, compared to the 5 % and 95 % quantiles (Q5 and Q95) of the CMIP6 ensemble ("CMIP6") and CMIP6 constrained by observations for temperatures according to the method of [12] ("CMIP6 cont.").

The scope of the comparison was to reject the simulations which fall outside the quantiles' interval [5 % - 95 %] of CMIP6 raw projections for both temperature and precipitation changes in either summer (JJA), or winter (DJF) or in both seasons, as those simulations are considered non-consistent enough with the CMIP6 projections to be retained. In Fig. 8 this “rejection zone” is colored in red. On the contrary, the zone corresponding to quantiles' interval [5 % - 95 %] of CMIP6 projections for both temperature and precipitation is colored in blue.

We also considered the discrepancies in changes due to the bias corrections methods: in Fig. 8 blue points refer to values issued from ADAMONT correction and green points to values issued from CDF-t method. The displayed changes in bias-corrected temperature and precipitation in Fig. 8 offer sometimes consistent differences according to the bias-correction method used. In Fig. 8, where blue points refer to values issued from ADAMONT correction and green points to values issued from CDF-t method, the rejection criterion must be verified simultaneously for both the CDF-t and ADAMONT methods in order for a GCM/RCM pair to be excluded.

According to all these criteria, it was decided to discard two simulations, namely the IPSLCM5-MR/WRF381P and CNRM-CM5/RACMO22E who did not respect the chosen requisites both with ADAMONT and CDF-t corrections in summer, as it is visible in Fig. 8. This led to the ultimate list of 17 pairs detailed in Table 1.

Despite the selection, it should be noted that the Explore2 dataset does not cover the upper tail of the CMIP6 temperature change distribution, particularly in summer. This results in a potential discrepancy in terms of projected warming and impacts in France with respect to other studies if based on global CMIP6 simulations. This discrepancy between global climate models and regional climate models for summer temperature in France needs to be taken into account in all studies on the risks associated with extreme heat and its consequences (evapotranspiration during heat wave, extreme soil drought, megafires, peak energy consumption for air conditioning etc.). One way to reduce this discrepancy is to consider Explore2–2022 data according to the warming level approach, in particular TRACC [4]. For worst-case scenario approaches, data coming from CMIP6 experiment can be considered as a supplement.

### 4.2 Bias correction

As is the case for the GCMs, the RCMs have systematic errors (or biases) in their output due to a variety of causes (see for example [13] for the European scale). The use of raw outputs in impact models or climate impact assessments can give unrealistic results. That is why the raw output of each selected GCM/RCM couple has been corrected by independently applying two statistical adjustment methods: ADAMONT [6] and CDF-t [7]. This way, from each raw GCM/RCM couple, we produced two distinct corrected scenarios, considered of equal quality. With each simulation being corrected and downscaled twice, the dataset provides two bias-corrected runs for each GCM/RCM selected couple, which allows to account for the uncertainty coming from the statistical method applied. Both bias-correction methods are improvements of the classic quantile-mapping approach. The ADAMONT method is a quantile mapping applied per season and conditioned on weather regimes [14]. The CDF‐t method is an extension of the quantile mapping to the non-stationary case. Both statistical methods make an adjustment relying on reference data taken as “truth”; to build the Explore2–2022 dataset, the reference was the SAFRAN reanalysis [8,15] on a Lambert conformal (regular) grid (EPSG 27572^15^) over France comprising 143×134 = 19162 points with a horizontal resolution of 8 km. These statistical adjustments thus allow a further refinement of the horizontal resolution of the climate projections, from the nominal resolution of 0.11° (approximately 12 km) in the EURO-CORDEX simulations to 8 km as the reference SAFRAN reanalysis.

A complete methodological comparison between the two bias-correction methods is beyond the scopes of this article. The reader is referred to [6] and [7] to infer their main features. For scopes related to Explore2–2022 dataset usage, corrections from the two methods are considered of same quality. Both methods have advantages and disadvantages.

Table 6 reports the mean residual bias with respect to SAFRAN of the two bias adjustment methods within the ensemble (spatial mean) in calibration-period (1976–2005) for temperature (tasAdjust) and precipitation (prtotAdjust) in winter (DJF) and summer (JJA).

**Table 6:** Mean residual bias w.r.t. SAFRAN of the two bias-correction methods (ADAMONT and CDF-t) within the ensemble (spatial mean) in calibration period (1976–2005) for temperature (tasAdjust) and precipitation (prtotAdjust) in winter (DJF) and summer (JJA). Values in brackets are the 5th and 95th quantiles in the ensemble.

tasAdjust	ADAMONT	CDF-t
**DJF**	-0.15 °C -0.30 °C; -0.04 °C]	-0.02 °C [-0.04 °C; 0 °C]
**JJA**	+0.39 °C [+0.33 °C; +0.47 °C]	0 °C [0 °C; 0.1 °C]

prtotAdjust	ADAMONT	CDF-t

**DJF**	-7.8 % [-13.47 %; -5.42 %]	-0.26 % [-0.32 %; +0.03 %]
**JJA**	-0.26 % [-1.73 %; +1.82 %]	-0.53 % [-0.54 %; -0.19 %]

The presence of a little residual bias in ADAMONT depends on the fact it has a supplementary level of uncertainty given by weather regimes, their representations in GCMs (which can vary with respect to those in RCMs) and their classification. Indeed, as shown in [16], the residual bias is sensibly reduced when it is separately considered within each weather regime. At the same time, the use of weather regimes has the advantage of allowing the method to adjust to their potential modified frequency in future climates.

Table 7 shows the residual bias in annual maxima of precipitation.

**Table 7:** Mean residual bias of the two bias-correction methods (ADAMONT and CDF-t) within the ensemble (spatial mean) for precipitation annual maxima. Values in brackets are the 5th and 95th quantiles in the ensemble.

Annual maxima of precipitation	ADAMONT	CDF-t
**year**	+6.5 % [0 %; 15 %]	-5.6 % [-6 %; -5 %]

We remind that, as shown in [5] and discussed is the “Data Description” section, the contribution due to bias correction methods for temperature and precipitation is found to be much smaller than that of the other sources of uncertainty.

### 4.3 Bias correction of precipitation and snowfall

Particular attention has to be given to the procedure of bias correction of daily snowfall (which corresponds to the variable prsnAdjust presented in Table 2) due to their impact on the hydrology of mountain basins. The two statistical methods for bias correction treat this variable in different ways.

**Fig. 9:**
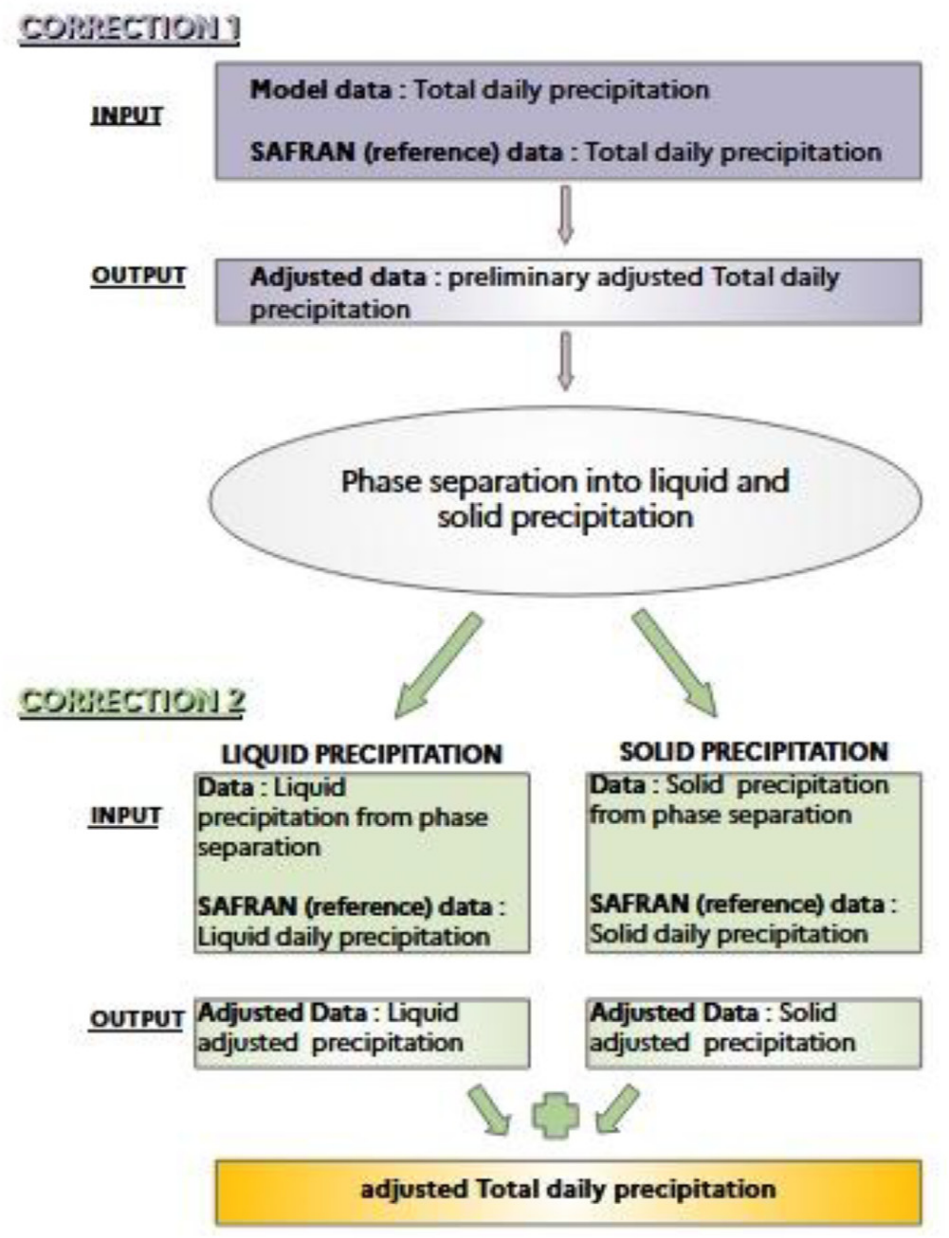
Illustration of the two-step correction approach implemented in ADAMONT for the treatment of snowfall.

ADAMONT uses a two-step correction. Initially, the algorithm corrects the total daily precipitation, as the sum of daily liquid and solid precipitation. At this point, a procedure of hourly disaggregation is applied to the daily corrected values of total precipitation; this is done by means of the observed hourly profile of an analogue day chosen in the SAFRAN reanalysis. The analogue day is chosen on the bases of the following criteria: for each day of simulation, an analogous day is selected from the SAFRAN data, as a day belonging to the same calendar month and weather regime [14] and chosen among the rainy days or non rainy days whether the raw data of daily precipitation is respectively greater or inferior to 1 mm. The hourly profile of total precipitation observed on the selected analogue day provides a profile for disaggregating the corrected daily values. Note that for a given day, the selected analogue day is the same for all points in the French territory. However, the hourly profile varies from one grid point to another (as does that of the analogue). Once the total precipitation has been disaggregated over 24 h, it is divided into solid and liquid precipitation based on the analogous hourly temperature profile, applying a threshold of 1.0 °C: in hours when the analogous temperature is greater than or equal to 1.0 °C, precipitation is considered as liquid, or as solid otherwise. Solid and liquid hourly precipitation is then separately summed within the day to obtain daily solid and daily liquid precipitation. A second correction is then applied to those daily quantities with respect to the reference daily liquid precipitation and daily solid precipitation in SAFRAN. All the analysis on the seasonal indicators shown in paragraph “data description” are done on the total daily precipitation prtotAdjust as the sum of liquid and solid precipitation after the second correction is applied. Fig. 9 illustrates the two-step correction of snowfall. More insights on the two-step correction in ADAMONT and its benefits can be found in [6].

**Fig. 10:**
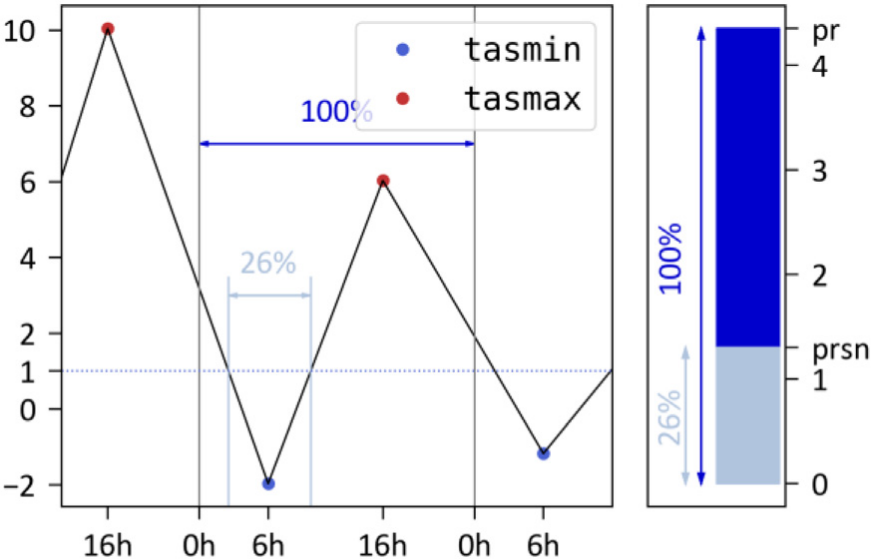
Illustration of the method used by CDF-t to calculate prsnAdjust snowfall from corrected data of total precipitation.

In CDF-t, daily snowfall is obtained from the ratio of hourly temperatures below 1 °C, applied to total precipitation. Fig. 10 illustrates this approach: from midnight to midnight (the temperature at midnight being given by a linear interpolation between the previous day's maximum temperature and the day's minimum temperature), 26 % of temperatures are below 1 °C, and therefore 26 % of total precipitation is in fact snow.

Table 8 summarizes the main differences in the treatment of snowfall by the two methods;

**Table 8:** Summary of the main differences in the treatment of snowfall by ADAMONT and CDF-t.

	ADAMONT	CDF-t
**Number of steps in the correction**	2	1
**SAFRAN (reference) data implied**	The sum of Liquid and Solid daily precipitation (first step correction) and separately Liquid daily precipitation and Solid daily precipitation (second step correction)	The sum of Liquid and Solid daily precipitation
**Diurnal temperature profile used for phase separation**	Hourly profile of an analogous day in SAFRAN dataset.	Interpolation between the previous day's maximum temperature and the day's minimum temperature
**Temperature threshold for phase separation**	1.0 °C	1.0 °C

### 4.4 The evapotranspiration

The potential evaporation (evspsblplot) is based on a Penman Monteith approach using the internationally recommended FAO^16^ formula with use of all adjusted variables of our dataset except for radiation where Hargreaves extrapolation (H coefficient of 0.175) has been considered, because of the issues with aerosol simulation in Regional Climate Models [17].

## Limitations

Several limitations should be taken into account when using this dataset

The overall size of the dataset, which may cause processing problems (990 GB), depending on users' IT resources.
Due to the use of a method by weather regimes, the ADAMONT correction method retains a bias over the calibration period [16].
The SAFRAN reanalysis [8,15] for France has flaws, particularly in terms of minimum and maximum surface temperatures. Those flaws have very little impact on hydrology; they require caution for users interested in studying temperature extremes.
The projection of temperatures and precipitations in the EURO-CORDEX simulations shows large discrepancies, particularly in summer, compared to global climate simulations over western Europe ([18,19]). Those discrepancies need to be taken into account in all studies on the risks associated with extreme heat. One way to reduce this discrepancy is to consider the Explore2-2022 set according to the warming level approach, in particular TRACC [4] and, for worst-case scenario approaches, data coming from CMIP6 experiment can be considered as a supplement.
Incomplete assessment of uncertainties related to climate models and internal variability particularly for estimating anthropogenic changes in the short and medium term: limited number of GCMs taken into account, only one member by GCM (low sampling of internal variability).

## Ethics Statement

The authors have read and followed the ethical requirements for publication in Data in Brief and confirmed that the current work does not involve human subjects, animal experiments, or any data collected from social media platforms.

## Credit Author Statement

Paola Marson: Software, Validation, Formal analysis, Investigation, Writing - Original Draft, Writing - Review & Editing, Visualization

Jean-Michel Soubeyroux: Conceptualization, Methodology, Investigation, Writing - Original Draft, Writing - Review & Editing, Supervision

Lola Corre: Methodology, Investigation, Writing - Review & Editing

Raphaëlle Samacoïts: Validation, Writing - Review & Editing

Eric Sauquet: Conceptualization, Methodology, Investigation, Writing - Review & Editing, Visualization, Supervision

Yoann Robin: Software, Validation, Data Curation, Writing - Review & Editing, Visualization

Mathieu Vrac: Writing - Review & Editing.

## Data Availability

IPSL Data catalogExplore 2 dataset: a set of regionalized and bias corrected climate projections for impact studies over France. (Reference data).

## Appendix Supplementary materials

Image, application 1

## Footnotes

2 https://www.drias-climat.fr/commandehttps://www.drias-climat.fr/.

3 https://data.ipsl.fr/catalog/sr/eng/catalog.search#/metadata/4aba213f-ad73-431d-b914-88ce1b42468d.

4 https://meteofrance.com/climadiag-commune.

5 https://climadiag-agriculture.fr/.

14 To judge the realism of GCM climate over Europe we followed the approach suggested by McSweeney [20], who defined a qualitative framework for evaluating CMIP5 models in order to select them for climate downscaling across multiple regions and subregions. McSweeney does not detail specific numerical metrics, but rather describes a qualitative selection process based on model performance in key climate processes such as large circulation patterns, storm trajectories, and local annual precipitation and temperature cycles.

## Footnotes

6 *DRIAS - les-futurs-du-climat :*www.drias-climat.fr.

7 The Explore2 project (2021-2024) is a national project launched in 2021 on a joint initiative of French scientists, the French Ministry of the Environment and funded by French Ecology Ministry and French Biodiversity Office, with the aim of updating knowledge on the impact of climate change on hydrology in France and supporting stakeholders (ministries, water agencies, local authorities, economic actors, …) in understanding and using data and results in order to build water management and adaptation strategies.

8 https://euro-cordex.net/.

9 https://entrepot.recherche.data.gouv.fr/dataverse/explore2.

10 “Trajectoire de Réchauffement de référence pour l’Adaptation au Changement Climatique”.

11 https://epsg.io/27572-to-4230.

12 https://euro-cordex.net/.

13 https://confluence.ecmwf.int/pages/viewpage.action?pageId=283549812.

15 https://epsg.io/27572-to-4230.

16 https://www.fao.org/4/X0490E/x0490e06.htm#fao%20penman%20monteith%20equation.
