# Supplementary material for: The Explore2–2022 climate projections dataset for impact studies over France
Source: Data Brief. 2026 Mar 9;66:112659. doi: 10.1016/j.dib.2026.112659 (PMC13011224; doi:10.1016/j.dib.2026.112659)
Supplement: Supplementary file 1 [file mmc1.zip › 20251208_Vsubmitted_Explore2data-paper_suppl_material.odt]

|  |
| --- |
|  |
|  |

Figure 1 suppl.: Ensemble matrices displaying the maps of relative differences (%) in cumulative winter DJF (top panel) and summer JJA (bottom panel) precipitation at the end of century (H3, 2070-2099) for the RCP8.5 scenario compared with the 1976-2005 reference period for each of the 17 GCM/RCM pairs in the Explore2-2022 ensemble. The rows correspond to GCMs, the columns to RCMs (ensemble, Explore2-2022, data corrected by CDF-t method). Hatching marks the grid points where the changes are non-significant with respect to the internal variability, according to the method applied in IPCC AR6 .

|  |  |
| --- | --- |
| Figure 2 suppl.: multi-horizon matrix displaying the spatialized distribution of the relative changes in winter DJF precipitation within the ensemble Explore2-2022 at three time horizon, H1: 2021-2050, H2: 2041-2070, H3: 2070-2099 with respect to the baseline period 1976-2005 under the RCP 8.5 scenario. At each grid point the minimum (left column), median (central column) and maximum (right column) value within the ensemble is shown. Hatches on the map of median changes is when for a grid point the accord in sign of the 80% of simulations is not reached.  Data displayed is corrected by the CDF-t method. | Figure 3 suppl.: multi-horizon matrix displaying the spatialized distribution of the relative changes in summer JJA precipitation within the ensemble Explore2-2022 at three time horizon, H1: 2021-2050, H2: 2041-2070, H3: 2070-2099 with respect to the baseline period 1976-2005 under the RCP 8.5 scenario. At each grid point the minimum (left column), median (central column) and maximum (right column) value within the ensemble is shown. Hatches on the map of median changes is when for a grid point the accord in sign of the 80% of simulations is not reached.  Data displayed is corrected by the CDF-t method. |

|  |  |
| --- | --- |
| Figure 4 suppl.: multi-scenario matrix displaying the spatialized distribution of the relative changes in winter DJF precipitation within the ensemble Explore2-2022 at the time horizon H3: 2070-2099 with respect to the baseline period 1976-2005 under the emission scenarios RCP2.6, RCP4.5 and RCP8.5. At each grid point the minimum (left column), median (central column) and maximum (right column) value within the ensemble is shown. Hatches on the map of median changes is when for a grid point the accord in sign of the 80% of simulations is not reached.  Data displayed is corrected by the CDF-t method. | Figure 5 suppl.: multi-scenario matrix displaying the spatialized distribution of the relative changes in summer JJA precipitation within the ensemble Explore2-2022 at the time horizon H3: 2070-2099 with respect to the baseline period 1976-2005 under the emission scenarios RCP2.6, RCP4.5 and RCP8.5. At each grid point the minimum (left column), median (central column) and maximum (right column) value within the ensemble is shown. Hatches on the map of median changes is when for a grid point the accord in sign of the 80% of simulations is not reached.  Data displayed is corrected by the CDF-t method. |

|  |
| --- |

Fig 6 suppl.: mosaic matrices referring to the summer (JJA) precipitation evolution displayed in multi-horizon matrix in Figure 3 suppl.
